# Human-animal chimeras for vaccine development: an endangered species or opportunity for the developing world?

**DOI:** 10.1186/1472-698X-10-8

**Published:** 2010-05-19

**Authors:** Anant Bhan, Peter A Singer, Abdallah S Daar

**Affiliations:** 1 McLaughlin-Rotman Centre for Global Health, University Health Network and University of Toronto, Toronto, Ontario, Canada; 2 McLaughlin-Rotman Centre for Global Health, University Health Network and University of Toronto, Toronto, Canada and Department of Medicine, University of Toronto, Toronto, Ontario, Canada; 3 McLaughlin-Rotman Centre for Global Health, University Health Network and University of Toronto, Toronto, Canada and Dalla Lana School of Public Health, University of Toronto, Toronto, Ontario, Canada

## Abstract

**Background:**

In recent years, the field of vaccines for diseases such as Human Immunodeficiency Virus (HIV) which take a heavy toll in developing countries has faced major failures. This has led to a call for more basic science research, and development as well as evaluation of new vaccine candidates. Human-animal chimeras, developed with a 'humanized' immune system could be useful to study infectious diseases, including many neglected diseases. These would also serve as an important tool for the efficient testing of new vaccine candidates to streamline promising candidates for further trials in humans. However, developing human-animal chimeras has proved to be controversial.

**Discussion:**

Development of human-animal chimeras for vaccine development has been slowed down because of opposition by some philosophers, ethicists and policy makers in the west-they question the moral status of such animals, and also express discomfort about transgression of species barriers. Such opposition often uses a contemporary western world view as a reference point. Human-animal chimeras are often being created for diseases which cause significantly higher morbidity and mortality in the developing world as compared to the developed world. We argue in our commentary that given this high disease burden, we should look at socio-cultural perspectives on human-animal chimera like beings in the developing world. On examination, it's clear that such beings have been part of mythology and cultural descriptions in many countries in the developing world.

**Summary:**

To ensure that important research on diseases afflicting millions like malaria, HIV, Hepatitis-C and dengue continues to progress, we recommend supporting human-animal chimera research for vaccine development in developing countries (especially China and India which have growing technical expertise in the area). The negative perceptions in some parts of the west about human-animal chimeras can be used as an opportunity for nurturing important vaccine development research in the developing world.

## Background

### The growing interest in chimeras

We need animal models that can be used to test vaccine candidates against neglected (and less neglected) tropical diseases such as malaria, dengue, HIV and Hepatitis C. These diseases cause morbidity and mortality in large numbers of people, with the developing world bearing the heaviest burden. Vaccine candidate testing in large non-human primates like chimpanzees is very costly, the number of animals available is usually small and there are concerns about inter-animal variability which affects data interpretation; and also most jurisdictions discourage research on large non-human primates due to ethical concerns. Translating laboratory findings directly into humans without adequate testing in animal models is risky, is frowned upon by regulatory agencies, and is not acceptable under current ethics guidelines. Human-animal chimeras provide an in-vivo system for these necessary studies [1], and are developed for this purpose. Human-animal chimeras are particularly important because they can be made to have components which resemble the human immune system or human liver for early, efficient and fast testing for efficacy of new vaccines; this is crucial because of the unique human tropism of these diseases. They can also be used for safety studies and to understand other effects of new technologies before they are tested in humans. As such, the creation of human-animal chimeras is more and more being seen as a valuable experimental tool that could revolutionize science and medicine [2]. They have been described as 'indispensable' for answering fundamental questions in stem cell and developmental biology' [3]. In this article, we focus especially on human-animal chimeras and some of the issues surrounding human embryonic stem cells (hESCs), which are used in their construction. We should note that human-animal chimeras of the type we discuss here are in essence xeno-transplantation models, and involve engraftment of human cells (such as blood forming progenitor cells, hepatocytes etc.) into specially conditioned mice.

Investments have been tracked for neglected disease research and development [4]. Evidence indicates that human-animal chimeras have an important role in testing new preventative vaccines not only for HIV, but also for diseases such as dengue virus infection and other disease [5] and for modeling the in vivo interaction of Plasmodium falciparum parasites and human hepatocytes (such studies would not be ethical to conduct in humans, and too expensive and complex in primate models) [6]. Producing better chimeric mice through research could lead to a useful tool for testing new malaria vaccine candidates, as well as in better understanding malaria pathophysiology. Recent research has raised hopes that better chimeric mouse models of infection with hepatitis- C virus can be produced [7]. Hepatitis C infects 3 to 4 million people worldwide annually (including a significant proportion in developing countries) adding to the existing global burden of approximately 170 million people (about 3% of the world's population) who are chronically infected with Hepatitis-C virus and at risk of progressing to severe and potentially fatal liver disease, Human-animal chimeras have been used in research for the past few years, and have been crucial to study diseases and developing therapies for them. For example, the SCID-hu mouse (a chimeric immunodeficient mouse with implanted human immune system components) model has been used to study the following human infections [8], many of which are neglected diseases:

a) Viral: HIV, Measles, Molluscum contagiosum, Human papillomavirus, Respiratory syncytial virus, Hepatitis B, Hepatitis C, Varicella zoster virus, Vesicular stomatitis virus, Cytomegalovirus, Herpes Simplex virus, Dengue Flavivirus, Human herpesvirus-6, Human herpesvirus-8 (Kaposi's Sarcoma), Epstein-Barr Virus, Enteroviral endocarditis.

b) Bacterial: Helicobacter pylori, Shigella, Pseudomonas (cystic fibrosis), Group A Streptococcus (impetigo), Salmonella, Chlamydia trachomatis, Neisseria meningitides

c) Parasitic: Entamoeba histolytica, Cryptosporidium parvum, Plasmodium falciparum, Schistosoma spp., Trichuris spp, Toxoplasma gondii

Further work carried out in recent years on human-animal chimeras has led to the development of specific and improved strains with applications for translational research [9].

Major funding to develop "humanized" mouse models for vaccine testing has been made available, for example from the Grand Challenges in Global Health initiative [10]. The US National Academy of Sciences has developed guidelines for the use of chimeras for research, including mechanisms for oversight at institutions which plan to conduct research on human-animal chimeras [11].

But alongside growing interest in the use of chimeras for human health there has been vocal political opposition in the West. A well known conservative US Senator from Kansas, Sam Brownback, for example, introduced the "Human Chimera Prohibition Act of 2005" to oppose the use of certain kinds of human-animal chimeras [12]. While the xenotransplant chimeras under discussion here may not necessarily fit the types of chimeras prohibited in that act, the conflation of definitions, confusion about terminology, and linkage to the general discussion about human embryonic stem cells mean that public, political and funding support for the development of human-animal chimeras generally may be compromised. This risk was well illustrated President George W. Bush's 2006 State of the Union address, where he clearly expressed opposition to "human-animal" hybrids. Bush asked Congress to "pass legislation to prohibit the most egregious abuses of medical research [including] creating human-animal hybrids," because "human life is a gift from our Creator" that should never be "devalued." [13] The situation may improve somewhat with the more pro-science attitude of the Obama administration, but it is too early to be sure that the negative attitudes will be reversed. Indeed, a Gallup poll conducted in the first part of 2009 in the USA shows the lingering adverse, confused and contradictory public perception of hESCs, which, as noted, are commonly used for development of human-animal chimeric mouse models for vaccine testing: approximately 80% of respondents in the poll favored some sort of restrictions on hESC research, and only 14% favored no restrictions; at the same time, the poll showed that 57% respondents viewed hESC research as "morally acceptable" [14].

In this commentary, we recognize there are legitimate ethical questions related to chimeras, which we review briefly below. At the same time, the main health benefits of chimeras may accrue in the developing world, where views on chimeras may be more welcoming than in the West, and where scientific capacity is growing. We propose that this concatenation of factors leads to an interesting strategic opportunity of focusing a niche on chimera research for human health in the developing world itself.

## Discussion

### Ethical concerns about chimeras

Ethical reservations around the creation of human-animal chimeras have revolved around the discomfort regarding the transgression of species boundaries [15], and scientists have been urged to exercise restraint in the creation of human-animal chimeras [16]. The fact that these animals are designed to have an immune system or other components such as hepatocytes exhibiting the characteristics of the human immune or other physiological system through the use of treated and differentiated hESCs, and (probably in the future) induced pluripotent stem (iPS) cells, has led to a number of questions. First, what is the position of these entities in the hierarchy of species? Secondly, what would happen if human cells migrated to the central nervous system of such a chimera, and there made connections with the animal's neurons (though this is a very remote possibility given the current status of the advances in science and the refinements made in the protocols used)? Would the chimera then become more "human" by developing human feelings and fears? Would it feel oppressed in a cage but unable to communicate its pain and anguish? These are questions that are particularly apt in the case of "neural chimeras". How much of the neurons would need to be replaced with human neurons before this was a real concern, if at all? It is not surprising, therefore, that some have argued that human-animal chimeras have an unclear moral stature. The May 2007 issue of the *American Journal of Bioethics *had several articles with divergent views by Western authors dwelling on this issue, questioning the moral status of, and possibility of feelings, in a human neuron mouse, and various opinions as to what extent such research should be permitted. Other arguments have focused on the possibility of breeding generations of human-animal chimeras and the uncertainty of the nature of the offspring produced. The NAS guidelines prohibit the breeding of human-animal chimeras, where there is a possibility of the human cells having contributed to the germ line.

A multidisciplinary working group of scientists and bioethicists in a 2005 commentary in *Science *unanimously rejected ethical objections to the development of human-non human primate chimeras based on the unnaturalness or crossing of species argument. They supported the US National Academy's stand that special review would be appropriate to address lingering concerns about cognitive and behavioral changes in the research animals in the specific cases of human-non human primate neural grafting [17]. In spite of these recommendations, controversy continues to plague the research into human-animal chimeras in the West [18]. The question remains as to whether these latter positions will prevail over the conservative ones.

### Taking into account the health needs of the developing world

If the development of human-animal chimeras were to be stopped or slowed down significantly because of such positions, not only would science be held back but it would be the developing world that would suffer most. Though the political opposition in countries like the USA has tended to dominate the public debate on the issues, the perspective of developing countries in the matter of animal human chimeras is absolutely critical.

Indeed, there is a precedence of delays in the development of life-saving technologies when the perspective of communities in developing countries is not taken into account: an analogy could be drawn to the field of rotavirus vaccine development. An earlier version of the rotavirus vaccine was removed from the market in the US in 1999 when a few children in the West developed intussusceptions, which postponed plans for research and subsequent introduction of rotavirus vaccines in developing countries to the detriment of thousands, if not millions, of children there suffering or dying from rotavirus associated diarrhea. Commenting on the issue, Weijer has argued, and we agree, that even though there was a small risk of intussusceptions in children getting the vaccine, the benefits of testing the vaccine in developing countries, given the disease burden, far outweighed the risk [19]. Subsequent experience has shown that rotavirus vaccines have high efficacy against rotavirus gastroenteritis, no increased risk of intussusceptions [20], and could help prevent substantial morbidity and mortality especially in developing countries. These rotavirus vaccines are very likely cost-effective, a key concern for health systems in developing countries, as illustrated by a paper looking at Mexico as a case study [21]. The authors found that introducing the pentavalent rotavirus vaccine in the national immunization program could significantly bring down rotavirus infection-related hospital admissions, medical visits and deaths and would be a highly cost-effective intervention according to the WHO Commission on Macroeconomics and Health Criteria.

Similarly, even though the creation of human-animal chimeras in research makes some people uncomfortable in the West, the benefits of creating such chimeras to accelerate vaccine development for disease that kill many more people in the developing world will likely be seen to be greater than the potential risks. If the attitudes in the West harden further, might the developing world itself supply a solution?

### Developing world perspectives on chimeras welcoming

Human-animal chimeras have been part of the cultural traditions in most parts of the world. This includes the West, where descriptions of human shape-changers, who changed shape yet remained human, were part of folklore in both Christian and Jewish traditions. An example is the description of the 'Proteus legend' by Homer as a prophet who could change shape to various forms including animals, if captured [22]. However, in current debates in the west, opposition to human-animal chimeras based on conservative political viewpoints and 'moral taboo argument(s)' [23] is quite vocal, and influences policy making in this area. Expanding the purview beyond Western views on human-animal chimeras reveals several perspectives that might provide useful insight. Hinduism, widely practiced in India, has several mythological creatures in its religious pantheon that are part human and part animal, or are animals with divine powers. These include Ganesha, the elephant-headed god and Hanuman, the monkey-god. The cow is considered sacred and is worshipped, and there are other animals, which are associated with particular gods and considered companions (for example, Durga-lion, Ganesha-rats, Shiv-snake). Many of these divine animals are part of mythological stories involving the victory of good over evil in the world, especially for the benefit of the human race. Chinese mythology has reference to *qilin or chi*, chimeras that symbolize Confucian values like kindness, compassion, peace and prosperity [24]. This chimera was considered to be non-violent, unlike chimeras in the Greek tradition (a source for Western philosophy), which were considered violent and sadistic [25]. Africa, a very diverse continent, has the use of folktales, where humans might acquire animalistic qualities, or insects that take on human qualities [26].

While chimeras also exist in Western mythology, the difference is that in places like India these are not just mythologies that were paid attention to in the past: they are part of the realities of everyday life to this day. The argument that such ideas may justify modern science and technology when applied to improve human health has been applied elsewhere, for example in the literature on xenotransplantation [27].

Besides the presence of chimeras in their mythology, developing countries like China and India have shown rapid recent growth in the field of biomedical research, including the field of chimera research with increased funding and progress both in the public and private sector. There is already research involving human-animal chimeras taking place in China [28,29] and there is a significant amount of research involving hESCells in India [30]. A comprehensive study to document the extent of research in more developing countries has not yet been performed, and the regulatory environments are evolving [31].

## Summary

### A way forward

So what can be done to avoid the slowing down of vaccine development because of conservative Western attitudes? One solution would be for a dialogue between those who hold differing views, but it is difficult to see how attitudes could be changed rapidly enough to make any difference. Another approach may be for the developing world, especially countries such as China and India, to make research involving human-animal chimeras a specific niche, and to support and fund it. This would be a way to move the scientific field forward, and to aid the development of solutions to important global health problems; this would supplement the excellent work happening in the west in this area, which is sometimes held up because of conservative attitudes to human-animal chimeras. An example for such funding, for the intended benefit of the developing world, already exists: The Bill and Melinda Gates Foundation has funded a project at Peking University, China, through its Grand Challenges in Global Health Initiative, to develop a "humanized" chimeric mouse model with an immune system and liver similar to humans for testing and development of potential HIV and HCV vaccine candidates [32], and provided support to develop a regulatory structure for the project [33]. This is in addition to the funding granted to two consortia based in the west (Europe and the USA) as part of the same initiative for addressing the same scientific challenge of developing (humanized chimeric mice) model systems to evaluate live attenuated vaccine candidates. Researchers in developing countries with relevant scientific capacity such as China and India should develop research projects with this perspective in mind, knowing that, so long as they fulfill scientific and ethical criteria (including humane treatment of the human-animal chimeras, careful planning and conduct of experiments as well as regulation through mechanisms such as Animal Care and Use Committees and Stem Cell Research Oversight Committees) for funding, they are likely to be successful. They can turn a negative Western attitude into an opportunity for the developing world.

## Competing interests

The authors declare that they have no competing interests.

## Authors' contributions

AB, PAS and ASD jointly participated in drafting the manuscript. All authors read and approved the final version of the manuscript.

## Authors' Information

AB is a researcher in bioethics and global health based in India, and works on a research project housed at the McLaughlin-Rotman Centre for Global Health, University Health Network and University of Toronto, Toronto, Ontario, Canada.

PAS is Professor of Medicine at the University of Toronto, and is Senior Scientist and Director of the McLaughlin-Rotman Centre for Global Health, University Health Network and University of Toronto, Toronto, Ontario, Canada. He is also the Chief Executive Officer(CEO) of Grand Challenges Canada.

ASD is a Professor of Public Health Sciences and of Surgery at the University of Toronto, and is Senior Scientist and Director of Ethics and Commercialization at the McLaughlin-Rotman Centre for Global Health, University Health Network and University of Toronto, Toronto, Ontario, Canada. He is also the Chief Science and Ethics Officer of Grand Challenges Canada.

## Pre-publication history

The pre-publication history for this paper can be accessed here:

http://www.biomedcentral.com/1472-698X/10/8/prepub
